# Supplementary material for: Examination of VOC Concentration of Aroma Essential Oils and Their Major VOCs Diffused in Room Air
Source: Int J Environ Res Public Health. 2022 Mar 2;19(5):2904. doi: 10.3390/ijerph19052904 (PMC8910502; doi:10.3390/ijerph19052904)
Supplement: Supplementary file 1 [file ijerph-19-02904-s001.zip › ijerph-1588072-supplementary.pdf]

International Journal of
Environmental Research and Public Health

Supplementary materials

Examination of VOC concentration of aroma essential oils and their major VOCs diffused in room air

Toshio Itoh [1], Yoshitake Masuda [1], Ichiro Matsubara [1], Junichirou Arai [2] and Woosuck Shin [1,*]

*Correspondence: w.shin@aist.go.jp

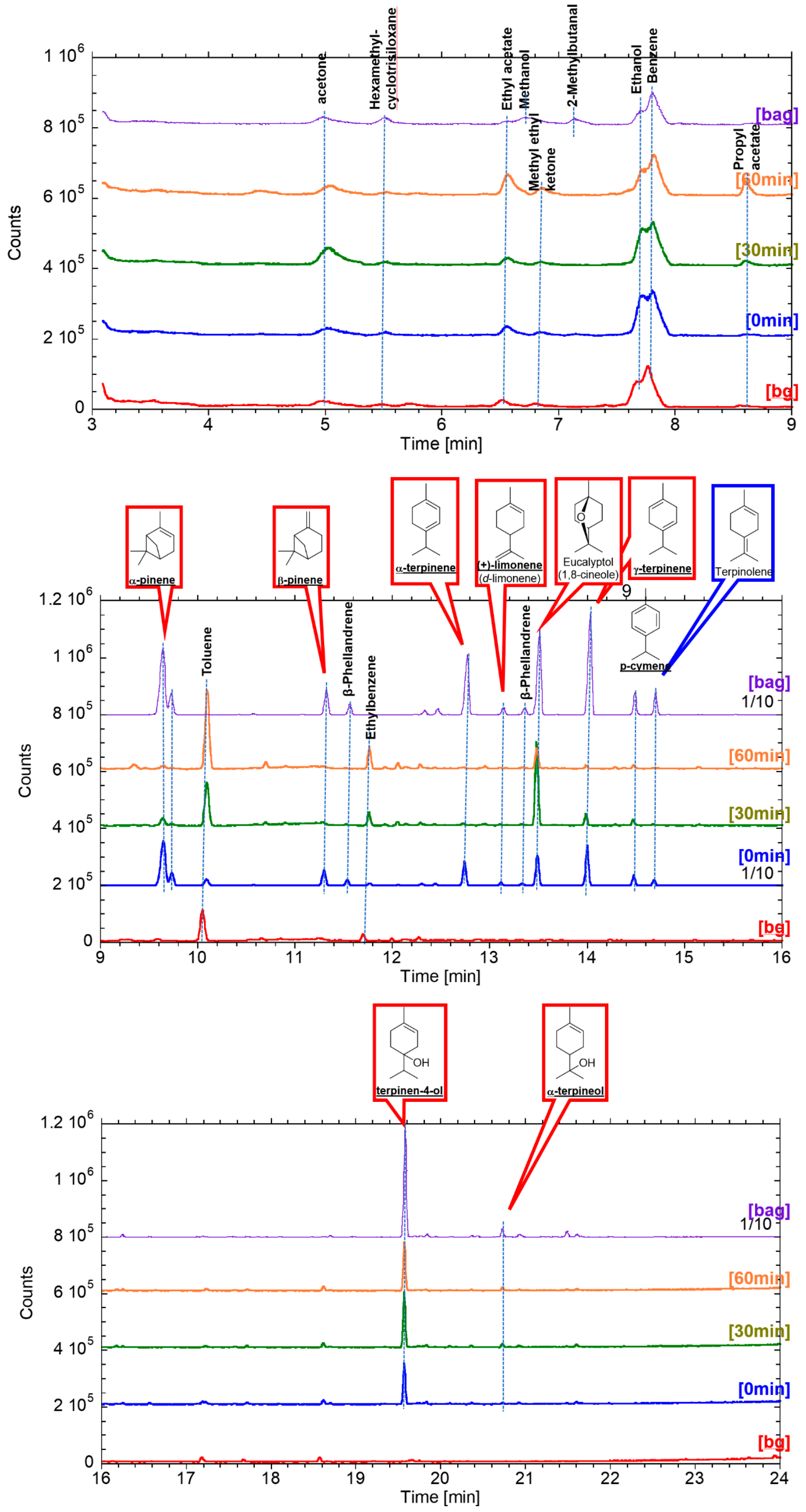

Figure S1. GC/MS chromatogram of tea tree.

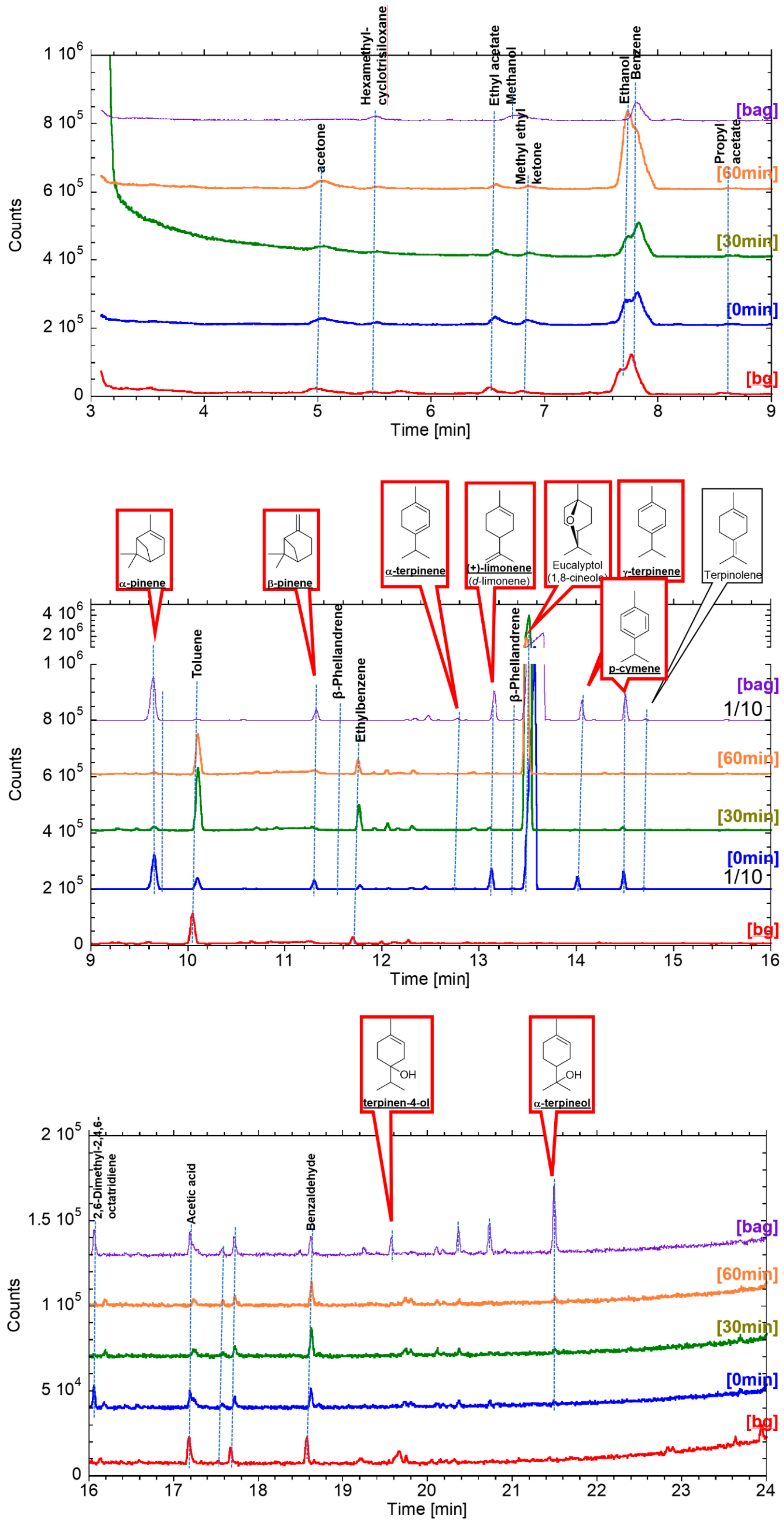

Figure S2. GC/MS chromatogram of eucalyptus.

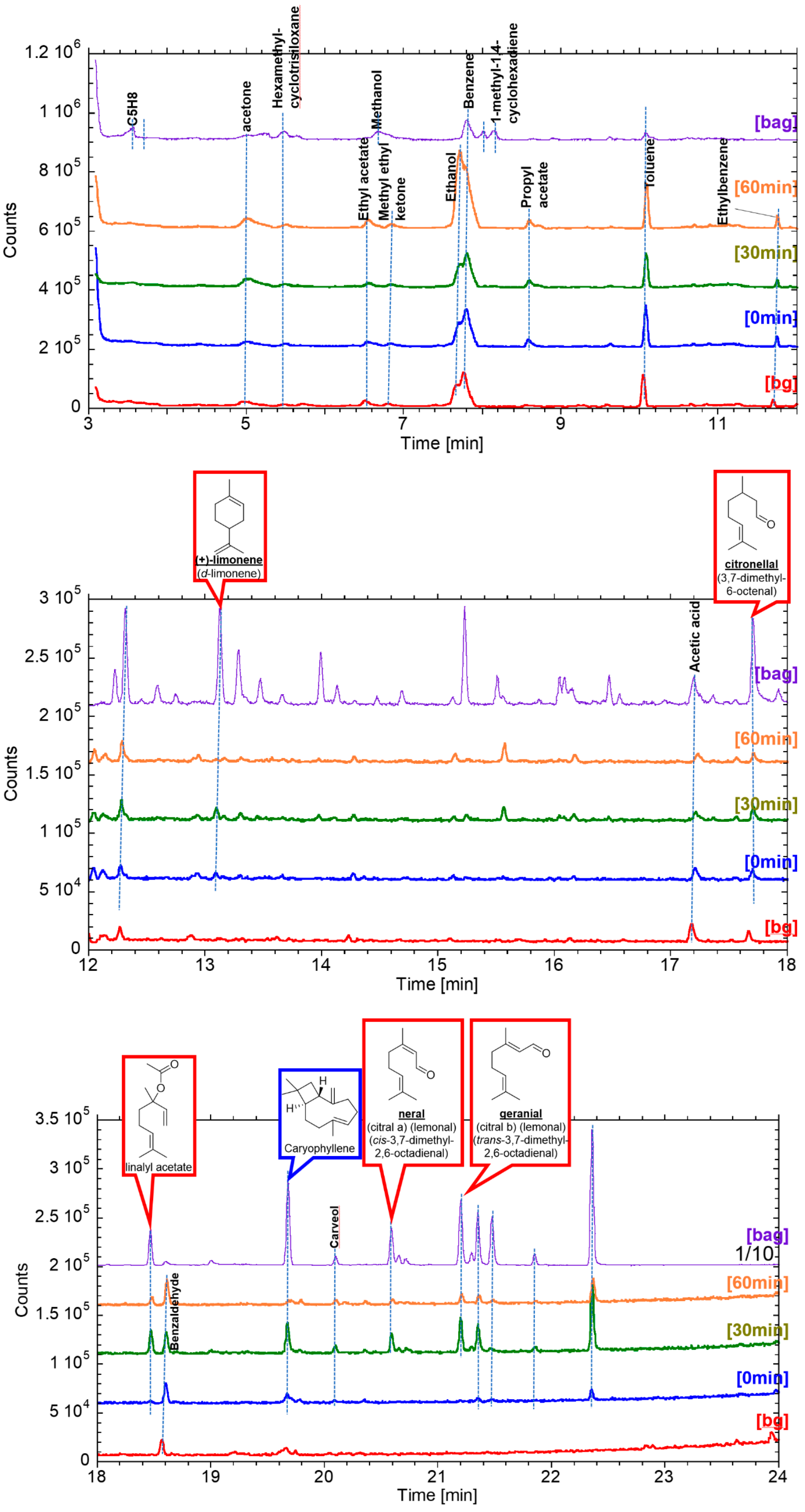

Figure S3. GC/MS chromatogram of melissa.

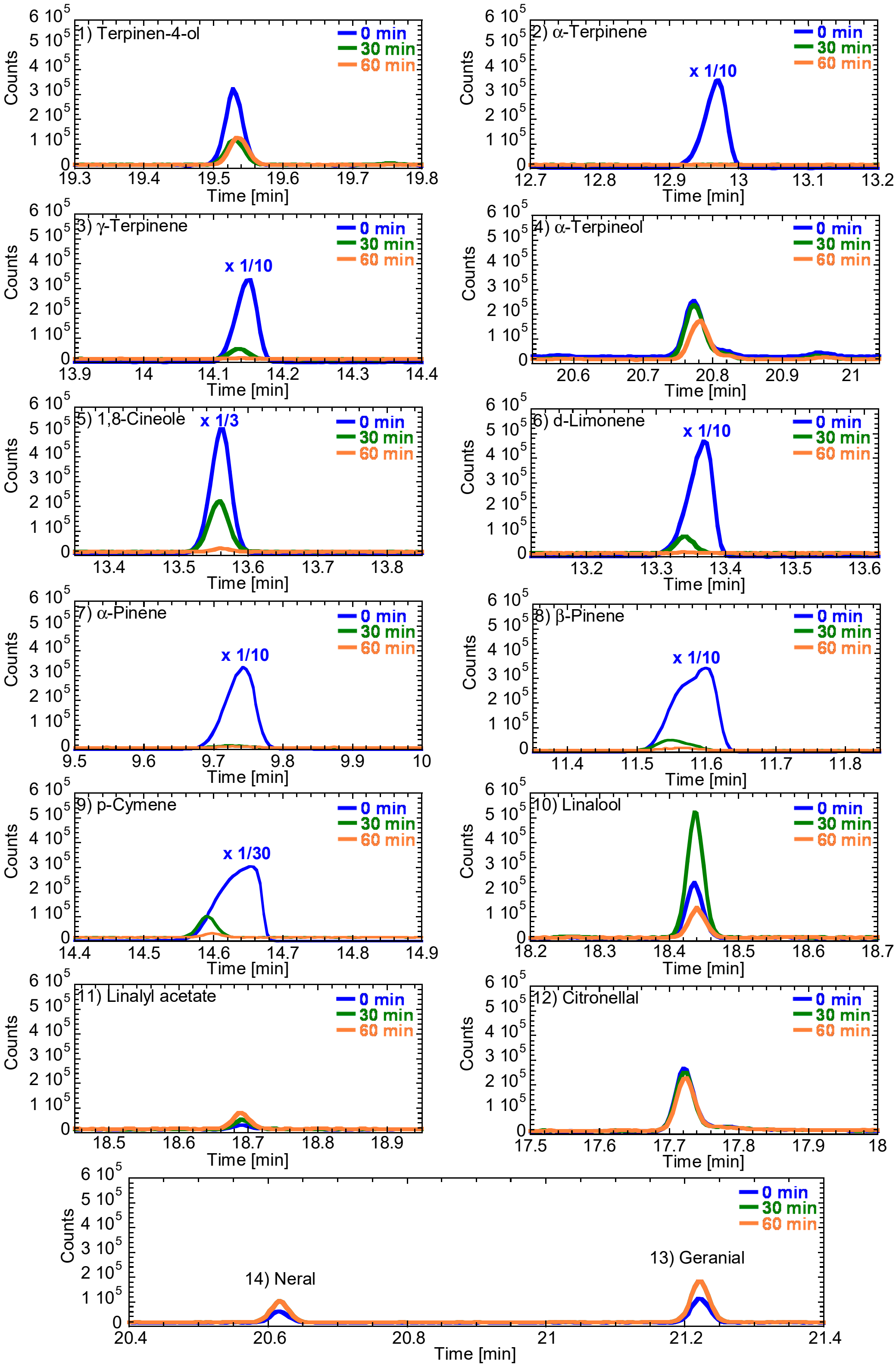

Figure S4. GC/MS spectra of 14 effective components.
